# Rehabilitative Good Practices in the Treatment of Patients with Muscle Injuries

**DOI:** 10.3390/jcm14155355

**Published:** 2025-07-29

**Authors:** Francesco Agostini, Alessandro de Sire, Nikolaos Finamore, Alessio Savina, Valerio Sveva, Andrea Fisicaro, Alessio Fricano, Umile Giuseppe Longo, Antonio Ammendolia, Andrea Bernetti, Massimiliano Mangone, Marco Paoloni

**Affiliations:** 1 Department of Anatomy, Histology, Forensic Medicine and Orthopedics, Sapienza University, 00185 Rome, Italy; francesco.agostini@uniroma1.it (F.A.); nikolaos.finamore@uniroma1.it (N.F.); alessio.savina@uniroma1.it (A.S.); valerio.sveva@uniroma1.it (V.S.); andrea.fisicaro@uniroma1.it (A.F.); alessio.fricano@uniroma1.it (A.F.); massimiliano.mangone@uniroma1.it (M.M.); marco.paoloni@uniroma1.it (M.P.); 2 Department of Medical and Surgical Sciences, University of Catanzaro “Magna Graecia”, 88100 Catanzaro, Italy; ammendolia@unicz.it; 3 Research Center on Musculoskeletal Health, MusculoSkeletalHealth@UMG, University of Catanzaro “Magna Graecia”, 88100 Catanzaro, Italy; 4 Fondazione Policlinico Universitario Campus Bio-Medico, 00128 Rome, Italy; g.longo@policlinicocampus.it; 5 Research Unit of Orthopaedic and Trauma Surgery, Department of Medicine and Surgery, Università Campus Bio-Medico di Roma, 00128 Rome, Italy; 6 Department of Biological and Environmental Sciences and Technologies (DiSTeBA), University of Salento, 73100 Lecce, Italy; andrea.bernetti@unisalento.it

**Keywords:** exercise, injury, muscle injuries, muscle lesions, good practice, rehabilitation

## Abstract

**Background:** The rehabilitative treatment of muscle injuries is mostly conservative, but it does not always follow precise protocols. Appropriate physiotherapy, exercises, and training are essential components of the rehabilitation and reconditioning of injured muscles. The purpose of this review is to assess the good rehabilitative practices in the treatment of patients affected by muscle injuries. **Methods:** We performed research on Medline and Cochrane Database. Guidelines focusing on the rehabilitative treatment of muscle injuries were evaluated for inclusion. Statements about non-rehabilitative treatments were also reported only for the guidelines that mainly focused on rehabilitative treatments. **Results:** Eight guidelines meeting the inclusion criteria were included in the review. Results were framed into a narrative overview. Two of them mainly focused on hamstring rehabilitation, the others focused on several muscular districts. **Conclusions:** Conservative treatment of muscle injuries is currently the gold standard, with good results in terms of both rehabilitation times and post-injury sports performance. However, there is not a complete agreement on the type of exercises and the timing of rehabilitation when these should be performed. More research is needed to draw conclusions about the use of physical therapy instruments and other rehabilitation approaches and techniques.

## 1. Introduction

Muscle injuries are a major cause of injury, representing between 10 and 55% of all acute sports injuries [1,2,3,4]. The most affected muscles are the rectus femoris, the muscles of the posterior thigh kinetic chain, and the gastrocnemius: These have intrinsic characteristics that predispose them to injury, such as biarticularity. However, injuries to other districts are also not uncommon [5,6,7].

A diagnosis can be made based on the medical history and clinical picture [8]. However, it is essential to describe the main characteristics of the injury, to develop an appropriate rehabilitation protocol, and to better predict the prognosis, the timing of the return to sport, and the possible risk of recurrence. For this purpose, imaging diagnostics are routinely used for an adequate diagnosis and the staging of muscle injuries, in particular an ultrasound and Magnetic Resonance Imaging (MRI) [9]. Ultrasound imaging allows the diagnosis of a structural lesion approximately 36–48 h after the trauma, due to the edematous hemorrhagic lesion generally observed 24–48 h after trauma [10]. Recent studies have also shown that the combination of MRI and ultrasound is superior to MRI alone in accurately measuring the extent of structural damage [11].

In the literature, there are several classifications based on imaging diagnostics, but we generally distinguish muscle injuries based on the traumatic mechanism. In the case of direct trauma, an external force is applied to the muscle, and the internal and external structures are compressed against each other. The injury results from various components, such as the intensity of the impact, the muscle involved, its contraction/relaxation state, and the traumatic moment. In the case of indirect trauma, the mechanism of injury is often related to a sudden passive stretching of the muscle during the contraction phase or to an excessively rapid contraction of the muscle starting from a state of complete relaxation. In this context, we distinguish between non-structural lesions (the most common, in which no real anatomical damage can be detected) and structural lesions [12].

Muscle injury classifications have evolved significantly to provide more precise descriptions and prognostic guidance for clinicians and researchers. It is currently recommended to use the classification system “Terminology and classification of muscle injuries in sport: The Munich consensus statement”. The Munich classificationdistinguishes between functional injuries (without evident structural damage) and structural injuries (with detectable structural damage), categorizing them by clinical and imaging findings. This classification has been used to standardize terminology and to guide the initial management strategies [13]. This classification is further detailed in Supplementary Table S1 [13].

However, the British Athletics Muscle Injury Classification (BAMIC), introduced in 2014, provides a grading system obtained by integrating advanced imaging modalities such as MRI. Unlike the Munich system, which focuses on clinical–functional differentiation, BAMIC emphasizes prognostic implications, and it is tailored to predict return-to-play timelines more accurately [14].

Supplementary Table S2 highlights the BAMIC framework, offering an advanced perspective on muscle injury characterization compared to the foundational Munich classification.

Muscle injuries are generally treated with a conservative approach with moderate success. Early return to activity is desirable [3]. The primary goal is to return the patient to a level of function comparable to pre-injury, with minimal risk of re-injury [15].

It has been observed that without an appropriate rehabilitation program that differentiates between the interventions available at different stages, the consequences of a muscle injury can lead to altered neuromuscular control, persistent muscle weakness, or reduced extensibility of the musculotendinous unit, partly due to residual scar tissue and partly due to the adaptive changes in the biomechanical and motor patterns of different movements [16].

Exercise interventions may help to prevent injuries [17]. A rehabilitation program should be accurately targeted to the tissue healing phase, and it should also correct the modifiable factors that may have contributed to the original injury [18]. This is achieved through the use of therapeutic exercise and manual techniques, such as joint and soft tissue mobilization. Appropriate physiotherapy, exercises, and training are essential components of the rehabilitation and reconditioning of injured muscles [8]. However, different types of exercise and different rehabilitative techniques have been suggested in the literature [19,20,21,22,23].

Other types of treatment are frequently used in clinical practice, such as instrumental physical therapies (e.g., cryotherapy, ultrasound, laser therapy, analgesic electrotherapy, endogenous thermotherapy, blood flow restriction training (BFR), and neuromuscular taping) and pharmacological/infiltrative interventions (e.g., non-steroidal anti-inflammatory drugs, platelet-rich plasma injections, antifibrotics, and supplements). While the efficacy of progressive exercise and initial protocols is supported by consolidated evidence and often recommended as the cornerstone of treatment, for many of these other therapies the scientific evidence is variable, with some opinions concurring and others discordant, highlighting the need for the definitive validation and standardization of protocols [1,2].

In this context, taking into account the recent rehabilitative guidelines [23,24,25,26,27,28,29,30], the present narrative review aimed to summarize and compare the recommendations for patients with muscle injuries to identify the best clinical practice in their management and to identify potential gaps or inconsistencies in the scientific literature.

## 2. Materials and Methods

### 2.1. Eligibility Criteria

We focused on Clinical Practice Guidelines (CPGs), defined by Field and Lohr in this way: “Clinical practice guidelines are systematically developed statements intended to assist both practitioners and patients in making decisions about appropriate healthcare for specific clinical situations” [24]. Only English-language guidelines were evaluated for inclusion. When several versions of the same CPG were present, the most recent one was chosen.

Guidelines focusing on patients with muscle injuries were evaluated for inclusion [13]. Muscle injuries were those defined by the Munich classification. There was no specific restriction for the lesion site: we evaluated both guidelines focusing on a specific muscle group and those focusing more generally on muscle lesions. There was no specific restriction for populations suffering from muscle lesions. The interventions under study were the rehabilitative protocols or treatments (physical therapies, exercise therapy, and other rehabilitative recommendations). We evaluated comparisons with other rehabilitative techniques, instrumental therapies, and pharmacological treatments for inclusion. Guidelines focusing on the following health outcomes were evaluated for inclusion: functional outcomes (e.g., return to sport, return to normal activity, or improvements in functionality questionnaires) and pain relief.

We did not include in our review guidelines focusing on the following: surgical or pharmacological treatments for patients with muscle lesions; rehabilitation after surgical treatment; and exercise therapy to prevent muscle lesions. The results were summarized in a narrative overview of the recommended treatments.

A pre-established protocol was registered on the International Prospective Register of Systematic Reviews (PROSPERO) with the registration code: CRD42025636441.

### 2.2. Information Sources and Search Strategy

Two different databases (Cochrane CENTRAL and Medline) were systematically searched, from their inception up to May 2025 using a specific search strategy, and our search was restricted to English-language articles. References for the included guidelines were also screened to find other potentially eligible studies. We also consulted a guidelines database (guideline.gov) and the websites of the main international scientific societies for further suitable articles. The database of guidelines and the websites were searched using an association of the MeSH terms “muscles”, “musculoskeletal diseases”, “sprains and strains”, “rehabilitation”, “exercise”, and “physical therapy modalities” and the terms “muscular”, “injury”, and “lesion” connected with different Boolean operators. See Supplementary Material S1 for the search strategy and for more details on the information sources.

### 2.3. Selection Process, Data Extraction, and Data Synthesis

After removing duplicates, two reviewers independently screened the records by title and abstract. The full texts of the articles that met the inclusion criteria were then independently evaluated for inclusion by the same two reviewers. Articles were included if both the reviewers considered them eligible. In cases of disagreement, a third reviewer (the same for both phases) was consulted to reach a consensus.

Once the screening phase was complete, the same two reviewers extracted data from the included studies independently using a customized Microsoft Excel template. The template included data on the title, authors, society, publication year, journal, multidisciplinarity, and the recommendations for rehabilitative and other treatments. Data on rehabilitative and other treatments have been framed in a narrative synthesis.

### 2.4. Bias Assessment

The Appraisal of Guidelines for Research & Evaluation II (AGREE II) tool was used to assess the risk of bias, and it is useful for evaluating the methodological rigor, transparency, and quality of the guidelines [23,24,25,26]. It is a 23-item tool comprising 6 quality domains (1—Scope and Purpose; 2—Stakeholder Involvement; 3—Rigor of Development; 4—Clarity of Presentation; 5—Applicability; and 6—Editorial Independence) and two global ratings. Each item and the two global rating items are rated on a 7-point scale (ranging from 1—strongly disagree to 7—strongly agree). Two reviewers independently assessed the risk of bias for each item, and the results have been synthesized into tables.

## 3. Results

### 3.1. Study Selection

A total of 539 studies were initially identified by searching the databases. One article was excluded as it was a duplicate, and 538 articles were screened by title and abstract by the reviewers. After this phase of screening, thirty articles were considered eligible and were retrieved for full-text assessment. One potentially eligible report was not retrievable, and it was excluded. Among the 29 articles evaluated as a full-text assessment, 26 were excluded with these reasons: treating a different topic (5 studies); not a rehabilitative intervention (5 studies); different population (2 studies); and not a guideline (14 studies). A list with the records excluded is reported in Supplementary Material S1.

At the end of the search, three guidelines were included in the review [1,2,30]. Two CPGs [27,29] were obtained by screening the references of one of the included studies [1]. Three guidelines [26,28,31] were obtained by searching the database of guidelines and websites. Among the results of the websites, one was excluded as it did not focus on rehabilitation (see Supplementary Material S1).

**Figure 1:**
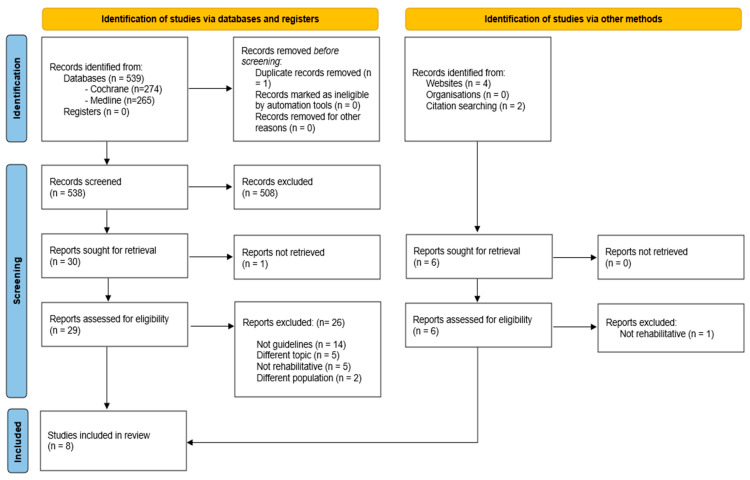
PRISMA flowchart.

Thus, at the end of the selection process, eight clinical practice guidelines were included in this review. Figure 1 reports the PRISMA flowchart and the selection process.

### 3.2. Study Characteristics

The main information on the guidelines included is summarized in Table 1.

In this table we have also reported information on multidisciplinary approaches. Two of them mainly focused on hamstring rehabilitation; otherwise, the others focused on more muscular districts [1,2].

A narrative synthesis of the main characteristics of the included guidelines is also reported:

- Paton B.M. et al. (2023) focused on the management of hamstring injuries. They gave directions for the selection of exercises, the progression of rehabilitation, and for the criteria to return to sport. In this document there is no mention of the level of the recommendations provided [1].
- FC Barcelona and the Aspetar Foundation [26] (a foundation located in Doha, Qatar, specializing in orthopedic and traumatological pathologies and sports medicine) collaborated in 2015 to develop a guideline which primarily focused on the diagnosis and treatment of the most common muscle injuries in professional footballers (both conservative and non-conservative). The research methodology used is not mentioned or explained, nor are any specific recommendations provided.
- Maffulli et al. [27] (2015) developed the guidelines of the Italian Society of Muscles Ligaments and Tendons (ISMuLT). These guidelines focused both on the diagnosis (both clinical and instrumental), classification, and treatment (surgical or conservative), and the treatment of muscle injuries. This document represents an update to the 2013 guidelines of the Italian Society of Muscles, Ligaments, and Tendons (ISMuLT) [29].
- The National Collegiate Athletic Association (NCAA) [28], in 2014, developed a document consisting of a set of guidelines for the management of muscle injury in sports in the collegiate athlete. They focused not only on the injury itself, but also on the psychological/motivational aspects of the athlete and on the equipment, from the perspective of 360-degree management. In this document there is no mention of the level of the recommendations provided.
- Valle et al. [30], in 2011, focused on all aspects of the management of the patient with the muscle injury, including the prevention of re-injury. They did not provide clear recommendations but attributed to every source they included a score based on the methodological value used in the writing of the article. In this way, a level of recommendation is indirectly attributed.
- The Association of Chartered Physiotherapists in Sports and Exercise Medicine (ACPSM) [31], in 2010, produced a guideline on the use of the PRICE protocol (Protection, Rest, Ice, Compression, and Elevation) in case of muscle injury. The document provides clear recommendations, as well as their level using GRADE.
- Heiderscheit et al. [2], in 2010, wrote a detailed description of the diagnostic and therapeutic pathway for muscle injuries involving only the hamstrings. No recommendations are provided, nor the level of the recommendations or the sources used.

### 3.3. Risk of Bias Assessment

The evaluation of distinct clinical practice guidelines using the AGREE II scoring criteria is reported in Table 2.

The first item of the overall assessment reached a mean score of 66.7%, ranging from 58.3% to 91.6%, which highlights the high heterogeneity in the methodological quality among the included studies. Examining the scores of each domain in more detail, Domain 4 (“Clarity of Presentation”) achieved the highest mean score of 88.5%. Domain 3 (“Rigor of Development”) received the lowest mean score of 30.3%. Nevertheless, both the reviewers would recommend the included guidelines, even with modifications (Overall Assessment, Item 2).

### 3.4. Synthesis of the Results

The main findings on rehabilitation treatments for muscular injuries are presented in the tables. To facilitate consultation, we have summarized the data obtained according to the following criteria: all the information on suggested exercises in Table 3.

More details:

(1) Isometric exercises are recommended by six CPGs reporting its usefulness in the post-acute phase [1,2,26,27,29,30]. One [26] suggests its potential effect in the initial phase of treatment.
(2) All CPGs [1,2,26,27,28,29,30], except from Beakley et al. [31], recommend eccentric exercise. However, Paton et al. [1] state that it should not be performed in the acute phase, although it is suggested in the advanced phase of rehabilitation.
(3) Concentric exercises are recommended by four guidelines [1,2,27,29]. Two of them recommend them in the post-acute phase [27,29].
(4) Isokinetic exercises are supported by three CPGs [1,27,30]. Two of them recommend them in the advanced phase [27,29].
(5) Plyometric exercises are supported by three CPGs [1,27,29] but only in the advanced phase of the rehabilitation.
(6) Stretching is recommended by six CPGs [2,27,28,29,30]. Another one reports uncertainty [1]. Two CPGs [27,29] suggest it in the post-acute phase. One [26] states that it should not be recommended in the early rehabilitation phase.

As reported in Table 4, concerning the recommendations for protocols and therapies we can affirm the following:

(1) All CPGs [2,26,27,29,30,31] but two [2,28] recommend PRICE.
(2) Two CPGs recommend POLICE [27,29].

Concerning therapies:

(1) One of the GCPs does not recommend NSAIDs [29]. Another one considers that there is uncertainty about their use [2].
(2) Two recommend laser therapy in the post-acute phase [27,29].
(3) Four CPGs recommend ultrasound therapy [2,27,29,30]. One [26] suggests it for DOMS, with Maffulli et al. [29] specifying the use of different modalities between the acute and post-acute phases.
(4) Three CPGs express uncertainty regarding the use of PRP [27,29,30].
(5) Two CPGs suggest neuromuscular taping [27,30].
(6) Three CPGs recommend the use of analgesic electrotherapy [2,27,29].
(7) Two CPGs recommend endogenous thermotherapy [27,29].

Concerning the other rehabilitative recommendations (see Table 5 for more details)

(1) Manual therapy is suggested by three CPGs [2,27,29]. Two of them support it in the acute phase [27,29].
(2) Core stability training is recommended in five CPGs [2,26,27,29,30]. Two of them specify its usefulness in the post-acute phase [27,29].
(3) Coordination and balance training is supported by five CPGs [2,26,27,29,30]. Two of them suggest it in the post-acute/advanced phase [27,29].
(4) Neurodynamic approach is recommended by four CPGs [1,2,27,29]. Two of them suggest it in the post-acute phase [27,29].
(5) Two CPGs recommend multitask exercises in an advanced phase of rehabilitation [27,29].
(6) Cardiovascular maintenance (aerobic exercise) is recommended by four CPGs [1,26,28,30].
(7) One recent CPG supports the use of hydrokinesis [1]. Another one reports uncertainty [27].

## 4. Discussion

The analysis of the literature shows that there is no consistency in the rehabilitative treatment of muscle injuries: The included CPGs report different perspectives on the topics covered, have been developed in different settings, or simply focus on a specific district. The CPGs included in our work often do not provide clear and targeted recommendations nor the level of evidence [1,2,26,27,28,29,30,31].

Two authors only focused on the rehabilitation of hamstring injuries without considering other muscle groups [1,2]. Despite these being injuries with a high incidence, the treatment of these muscle groups in athlete patients might not be considered the rule of thumb for the general population [32].

The AGREE II instrument highlights significant heterogeneity among the included guidelines’ domains. More details: The lowest mean score was achieved by Domain 3 (Rigor of Development), and Domain 5 (Applicability) also did not perform well. This may be related to the publication year, given that most of the guidelines were published over 10 years ago. Furthermore, the scores for these domains may also be influenced by the fact that some items are not entirely applicable to physiotherapy guidelines: items 19 (facilitators and barriers) and 21 (monitoring and auditing) relate to whether or not the treatment is carried out. It should be highlighted that only one guideline has been published in the last five years, with the oldest one being published in 2010 [1]. Multidisciplinarity is commonly found in the guidelines included, as also suggested in the literature [33].

A characteristic of the included evidence is that in several cases professional athletes are used as reference groups [1,26,28]. This population is not at all representative of the general population in terms of biometric indices, muscle mass, athletic ability, lifestyle, adherence to treatment, and finally, in terms of the possibility of being personally monitored by a medical staff.

Rehabilitation goals for athletes are geared toward the demands and capabilities required for competition, which are clearly higher than those of the general population aiming to return to normal daily activities. Indeed, specific exercises such as eccentric, isokinetic, and plyometric strengthening are often emphasized, with the goal of achieving full strength and adequate function for athletic performance. This implies a level of strength and specific muscle function not typically required in the non-athletic population. This level of detail in performance monitoring is generally absent in injury management for the non-athletic population, due to the unique physiological and performance demands, the pressure environment, and the preferred clinical sports diagnosis and monitoring protocols [1,2,26,27,28,29,30,31].

Regarding the types of exercises to be performed, there is no complete agreement on which exercises to do or in which phase of rehabilitation they should be performed (especially for stretching and isokinetic exercises). This could partially be explained by both the evolution of rehabilitation and the different populations of individuals: comparing the two CPGs on hamstrings, the most recent one reports more uncertain results for isokinetic and plyometric exercises and stretching [1]. Isometric and eccentric exercises are the most supported by the included CPGs, with some differences in the phases of rehabilitation. However, it should be highlighted that the stages of rehabilitation are categorized differently between the CPGs, and this could explain the differences in the suggested timing. No firm conclusions can be drawn about stretching. Its role in injury prevention is uncertain in the literature: [34] it is thought to improve muscle flexibility and reduce muscle pain in patients with hamstring injuries, [32,35] but there are inconsistencies. The authors believe that CPGs should better analyze the role of different types of stretching, as they may have different properties in the muscle [36]. When found, plyometric exercise is supported at an advanced phase, as these exercises depend on the elastic properties of the muscles: in fact, in line with the literature, they are mainly used for training [37].

All the CPGs facing core stability and aerobic exercise utility (defined as cardiovascular maintenance in the article) support their effectiveness, in line with what is known in the literature [38,39]. Also, one author [1] suggested that rehabilitation prescription should be individualized, but highlighted the lack of evidence regarding the selection criteria, timing, and optimal load dose for rehabilitation, as well as monitoring and testing to determine a safe rapid progression in rehabilitation and safe return to sport.

This method, also treated by other authors, [27,30] although not with any clear recommendation, is considered effective in reducing pain in the context of injuries of different muscle districts.

It is necessary to highlight some methodological and clinical issues. First of all, muscle injuries represent an extremely heterogeneous clinical field, not only in terms of the type and location of the muscle involved, but also in terms of the degree of injury (e.g., type I, II, or III according to radiological classifications), the stage of development (acute, subacute, or chronic), the characteristics of the patient (professional athlete vs. sedentary individual), and the pathogenic mechanisms (direct trauma, indirect trauma, overuse, etc.). Added to this heterogeneity is significant variability in the individual biological response to injury and treatment: tissue repair times, scar tissue quality, and tolerance to therapeutic load can differ widely between patients, even with the same injury pattern.

Pharmacological treatments are not present in all the guidelines as we mainly focused on rehabilitation; however, no consensus can be found on the use of non-steroidal anti-inflammatory drugs (NSAIDs) or for platelet-rich plasma (PRP) injection. However, the use of these treatments is controversial in the literature: despite the pain-killer effect, it has been reported in the literature that NSAIDs might interfere with the healing process, with an incomplete/delayed healing. Inflammation is a complex physiological response to tissue damage, aimed at clearing cellular debris, promoting angiogenesis, and initiating scar tissue formation. By suppressing inflammation, NSAIDs may potentially delay or impair muscle healing, resulting in a slower recovery and an increased risk of re-injury. While NSAIDs can provide symptomatic relief from pain and inflammation, their potential adverse effects on the healing process warrant careful consideration [40].

Some authors have paid attention to the use of physical energies in the therapeutic approach to muscular injuries but with some shortcomings [13,41,42,43,44]. The mechanisms of action and the biological effects of these medical devices are explained, but the application protocols are not specified with regard to the number of sessions, the total duration of treatment, and the phases of the healing process that are susceptible to these treatments. This lack in the literature probably reflects the extreme heterogeneity of the therapeutic protocols in the field of muscular injuries. Analgesic electrotherapy is suggested, although evidence supporting its efficacy remains limited [2,27,29].

Moreover, rehabilitation is constantly changing, leading to new protocols and competencies. There is no reference to these new rehabilitative approaches in the guidelines included. As previously reported, most of the guidelines included were published more than 5 years ago. Almost all the CPGs support the PRICE or the POLICE protocols, but other protocols have been developed or are emerging in the treatment of muscle lesions.

For example, Dubois et al. in 2019 [45] published in the British Journal of Sports Medicine (BJSM) the PEACE and LOVE protocol for the management muscle injuries. PEACE (Protection, Elevation, Avoid Anti-inflammatories, Compression, and Education) focuses on the acute phase of injury, while LOVE (Load, Optimism, Vascularization, and Exercise) addresses the recovery phase. This protocol represents an evolution from previous approaches such as RICE (Rest, Ice, Compression, and Elevation) and POLICE (Protection, Optimal Loading, Ice, Compression, and Elevation), for a more comprehensive and patient-centered rehabilitation. Despite its growing popularity, the PEACE and LOVE protocol has not yet been universally adopted into the official guidelines of major global medical institutions [45].

Regarding cryotherapy, which is universally recognized and included in the PRICE or the POLICE protocols, it is interesting to highlight the findings of Bleakley et al. The authors emphasize that the clinical efficacy of ice application in terms of functional recovery, edema reduction, and accelerated healing times is limited. Clinically, the use of ice must be individualized according to the type and location of the injury, considering that optimal analgesia can be achieved with ice for 5–15 min. Despite this, in everyday clinical practice, the use of ice seems to provide empirical benefits [31].

Manual therapy is recommended by three of the sources analyzed, with conflicting opinions and conflicting evidence. Although massage therapy has been suggested in the management of acute muscle injuries, evidence supporting its use for healing and recovery is generally lacking. There is conflicting evidence regarding the positive effect of massage therapy on muscle activity and hamstring flexibility in healthy adults, and there is no evidence regarding its effect on healing and recovery following an acute muscle injury. Despite the lack of robust evidence for direct healing, manual therapy in the form of specific massages is recommended in the early phase (acute management, 2–3 days after injury) to promote drainage of uncompromised tissues near the injury site and improve the disposal of inflammatory catabolites, thus serving as an adjuvant. However, it is necessary to clarify that this is a highly operator-dependent treatment, whose effectiveness is closely linked to the experience, training, and clinical sensitivity of the therapist [1,2,27,29,31].

Electroanalgesia is recommended as one of the instrumental physical therapy modalities to be used in the early phase of muscle injury management. This indication suggests a potential role in acute pain control. However, there is a lack of robust evidence, especially with regard to healing and recovery from acute injuries. There is a contrast and lack of uniform consensus among experts, with differences in global clinical practice and a still limited evidence base in this specific area [1,2,29,31].

There are also several treatments, not included in the guidelines due to limited evidence, that are sometimes used in clinical practice for the treatment of muscle injuries and seem to have promising results. Several authors [44,46,47,48] have looked at different approaches such as PRP, cortisone or local anesthetic injections, showing a certain degree of uncertainty in this regard. Williams et al. focused their work on the use of neuromuscular taping in sports injuries [49]. The effect of platelet-rich plasma on muscle repair is still under investigation, and the scientific evidence supporting its efficacy remains limited and of low quality [50].

Trofa et al. [51], in 2020, analyzed several non-surgical modalities used by athletes to improve performance or prevent, treat, and rehabilitate musculoskeletal injuries. They analyzed relevant publications regarding Kinesio taping, sports massage therapy, acupuncture, cupping, and blood flow restriction (BFR) from 2006 to 2019. There is some low-level evidence that suggests Kinesio taping for athletes with acute shoulder symptoms and acupuncture for carpal tunnel syndrome and as an adjuvant treatment for lower back pain. There is a need for higher quality research to better clarify the effect of sports massage therapy on sports performance, recovery, and musculoskeletal conditions generally. Cupping can be an effective, low-risk option treatment for non-specific musculoskeletal pain [51,52]. Comparative studies of BFR with non-BFR controls suggest it may increase muscle mass strength and endurance for people undergoing rehabilitation or sport-specific training, mimicking a low-oxygen environment during exercise. Cognetti et al. [53], in 2022, analyzed BFR therapy, and they concluded that BFR stimulates muscle hypertrophy via a synergistic response to metabolic stress and mechanical tension, with supplemental benefits on cardiovascular fitness and pain. However, the existing evidence is still emerging, and more robust studies are needed to fully validate its efficacy and standardize protocols [53]. Focused shockwave therapy (SWT) currently lacks sufficient studies in humans for its use in muscle regeneration. Likewise, hyperthermia and capacitive-resistive diathermy show low levels of evidence for their use in muscle injuries [54,55]. Neuromuscular taping has been considered by two CPGs, with uncertain results [27,30].

The use of Curcumin and Boswellia has shown promising results for their anti-inflammatory and analgesic properties, but further studies are necessary to confirm these findings [56,57,58].

Antifibrinolytics (TGF-1), specifically Losartan as an angiotensin II antagonist or Suramin, have only shown effects in experimental animal models [59,60]. The use of Actovegin, which is supported by studies with low levels of evidence, remains controversial [61]. Finally, the supplementation with vitamin D, omega-3 fatty acids, and amino acids could stimulate protein synthesis and counterbalance the loss of muscle mass during inactivity [62,63,64,65,66].

Furthermore, telerehab [67,68,69,70].

We acknowledge that our article is not free from limitations. Firstly, due to the study design, it was impossible to perform a meta-analysis. Moreover, one of the included guidelines has been published recently, though the others are ten or more years old. While it highlights a gap in this field, it may also influence our results, as important advances in diagnostic tools and more high-performance devices can help to further personalize treatments. Future guidelines may provide more specific recommendations for the rehabilitation of lesions of different grades. Furthermore, only a qualitative synthesis was possible, as the guidelines included give indications about what is recommended or not; while these results are important, they could be less interpretable in a research context, as they do not provide information on numerical changes. Another limitation is that most of the included studies provide recommendations with no specifics about the individual exercises, only discussing them in categories. Future guidelines should focus on more targeted treatments to achieve a greater standardization of results.

## 5. Conclusions

Taken together, findings of this narrative review show that conservative rehabilitative treatment remains the recommended therapeutic approach for most muscle injuries. However, there is no universal agreement on the type of exercise or the timing of administration. The existing evidence is often heterogeneous, with protocols that are not comparable in terms of application methods or outcome criteria. However, additional treatment modalities, including the use of physical therapies and infiltrative techniques, require further investigation. Despite their inclusion in some recommendations, specific parameters and treatment protocols are often insufficiently detailed in the literature.

Management should be personalized and progressive, aiming to ensure optimal recovery while minimizing the risk of re-injury. This requires consideration of both the characteristics of the injury and the individual characteristics of each patient. High-quality studies are still needed to provide stronger scientific evidence, which is essential for developing clinical guidelines and improving the standard of care for muscle injury management.

## Figures and Tables

**Table 1:** Characteristics of the main included guidelines.

Guideline	Authors/Society/Year	Multidisciplinarity	Hamstring
London International Consensus and Delphi study on hamstring injuries part 3: rehabilitation, running and return to sport [1]	Paton B.M. et al., 2023	Yes	Yes
Muscle injuries clinical guide 3.0 [26]	FC Barcelona, Aspetar, 2015.	Yes	No
Muscle injuries: a brief guide to classification and management [27]	Maffulli N. et al., 2015.	Yes	No
Sport medicine handbook: Guidelines [28]	National Collegiate Athletic Association, 2014.	Yes	No
ISMuLT guidelines for muscle injuries [29]	Maffulli N. et al., 2013.	Yes	No
Clinical practice guide for muscular injuries: epidemiology, diagnosis, treatment and prevention [30]	Valle X., 2011.	Yes	No
Acute management of soft tissue injuries: Guidelines [31]	Bleakley C.M. et al., 2010.	No	No
Hamstring strain injuries: recommendations for diagnosis, rehabilitation, and injury prevention [2]	Heiderscheit B.C. et al., 2010.	Yes	Yes

**Table 2:** Domain scores and overall assessment of Clinical Practice Guidelines using the AGREE II instrument.

CPG	Domain 1	Domain 2	Domain 3	Domain 4	Domain 5	Domain 6	Overall 1	Overall 2	
								R1	R2
Paton, 2023 [1]	91.6%	44.4%	52.0%	91.6%	35.4%	75.0%	83.3%	1	2
Valle, 2015 [26]	80.55%	44.4%	13.5%	88.8%	41.7%	37.5%	58.3%	2	2
Maffulli, 2015 [27]	80.5%	30.5%	17.7%	80.5%	18.7%	41.6%	58.3%	2	2
NCAA, 2014 [28]	83.3%	72.2%	26.0%	91.7%	27.1%	16.7%	66.7%	2	2
Maffulli, 2013 [29]	80.5%	52.7%	21.9%	88.9%	20.8%	20.8%	66.7%	2	2
Valle, 2011 [30]	88.8%	55.5%	14.6%	91.6%	33.3%	33.3%	58.3%	2	2
Bleakley, 2010 [31]	97.2%	58.3%	73.9%	80.5%	35.4%	83.3%	91.7%	1	2
Heiderscheit, 2010 [2]	80.5%	47.2%	22.9%	94.4%	37.5%	37.5%	58.3%	2	2

Abbreviations: CPG = Clinical Practice Guidelines; R1 = Reviewer 1; and R2 = Reviewer 2. Domain 1 (Scope and Purpose); Domain 2 (Stakeholder Involvement); Domain 3 (Rigor of Development); Domain 4 (Clarity and Presentation); Domain 5 (Applicability); and Domain 6 (Editorial Independence).

**Table 3:** Recommendations for different types of exercise.

	Isometric Exercise	Eccentric Exercise	Concentric Exercise	Isokinetic Exercise	Plyometric Exercise	Stretching
Paton B.M., 2023 [1]	R	R	R	R	R	I
Valle, 2015 [26]	R	R				R
Maffulli, 2015 [27]	R	R	R	R	R	R
NCAA, 2014 [28]		R				R
Maffulli, 2013 [29]	R	R	R	R	R	R
Valle X., 2011 [30]	R	R				R
Bleakley, 2010 [31]						
Heiderscheit, 2010 [2]	R	R	R			R

**Table 4:** Recommendations for protocols or specific therapies.

	PRICE	POLICE	NSAID’s	Laser Therapy	US Therapy	PRP Injections	Neuromuscular Taping	Analgesic Electrotherapy	Endogenous Thermotherapy
Paton B.M., 2023 [1]									
Valle, 2015 [26]	R								
Maffulli, 2015 [27]	R	R		R	R	I	R	R	R
NCAA, 2014 [28]									
Maffulli, 2013 [29]	R	R	NR	R	R	I		R	R
Valle X., 2011 [30]	R				R	I	R		
Bleakley, 2010 [31]	R								
Heiderscheit, 2010 [2]	R		I		R			R	

**Table 5:** Other rehabilitative recommendations.

	Manual Therapy	Core Stability	Coordination Balance	Neurodynamics	Multitask Exercise	Cardiovascular Maintenance	Hydrokinesis
Paton B.M., 2023 [1]				R		R	R
Valle, 2015 [26]		R	R			R	
Maffulli, 2015 [27]	R	R	R	R	R		I
NCAA, 2014 [28]						R	
Maffulli, 2013 [29]	R	R	R	R	R		
Valle X., 2011 [30]		R	R			R	
Bleakley, 2010 [31]							
Heiderscheit, 2010 [2]	R	R	R	R			

## Data Availability

No dataset available due to the study design.

## Supplementary Materials

The following supporting information can be downloaded at: https://www.mdpi.com/article/10.3390/jcm14155355/s1, Supplementary Material S1: Search Strategy; Supplementary Table S1: Classification of muscle injuries according to Mueller-Wohlfahrt H.W. et al., 2012. [13]; Supplementary Table S2: Classification of muscle injuries according to British Athletics Muscle Injury Classification (BAMIC).
