# Supplementary material for: Rehabilitative Good Practices in the Treatment of Patients with Muscle Injuries
Source: J Clin Med. 2025 Jul 29;14(15):5355. doi: 10.3390/jcm14155355 (PMC12347294; doi:10.3390/jcm14155355)
Supplement: Supplementary file 1 [file jcm-14-05355-s001.zip › jcm-3729831 Supplementary Material S1.pdf]

Search Strategy:

Medline

((((("Muscles"[Mesh] AND "Wounds and Injuries"[Mesh]) OR "Sprains and Strains"[Mesh]) AND "Rehabilitation"[Mesh]) OR "Sprains and Strains/rehabilitation"[Mesh]) OR ("Muscles/injuries"[Mesh] OR (“muscle injur*” [TIAB] OR “muscle tear*” [TIAB] OR “muscle lesion*” [TIAB] OR “muscular injur*” [TIAB] OR “muscular lesion*” [TIAB] OR “muscular tear*” [TIAB] OR “musculoskeletal injur*” [TIAB] OR “musculoskeletal lesion*” [TIAB])) AND ("Rehabilitation"[Mesh] OR "Exercise Therapy"[Mesh] OR "Physical Therapy Modalities"[Mesh] OR "Injections"[Mesh] OR (rehabil* [TIAB] OR (((Endurance [TIAB] OR Resistance [TIAB]) AND training* [TIAB]) OR “muscle stretch*” [TIAB] OR (exercis* [TIAB] AND (plyometric [TIAB] OR concentric [TIAB] OR eccentric [TIAB]))) OR (“Physical Therap*” [TIAB] OR "Magnetic Field Therapy" [Mesh] OR "Cryotherapy" [Mesh] OR "Ultrasonic Therapy" [Mesh] OR "Diathermy" [Mesh] OR ("Extracorporeal Shockwave Therapy"[Mesh] OR ESWT [TIAB] OR FSWT [TIAB]) OR (Tecar [TIAB] OR “Tecar therapy” [TIAB]) OR “ultrasound therap*” [TIAB]) OR (“injecti*” [TIAB] OR “injective therapy” [TIAB] OR ("Hyaluronic Acid"[Mesh] OR "Platelet-Rich Plasma"[Mesh]))))) AND (guidelines as topic[MeSH:noexp] OR practice guidelines as topic[MeSH:noexp] OR Health Planning Guidelines[MeSH:noexp] OR practice guideline[MeSH:noexp] OR clinical protocols[MeSH:noexp] OR Consensus[MeSH:noexp] OR "Consensus Development Conference"[PTYP] OR "Consensus Development Conference, NIH"[PTYP] OR "Consensus Development Conferences as Topic"[MeSH:noexp] OR "Consensus Development Conferences, NIH as Topic"[MeSH:noexp] OR critical pathway[MeSH:noexp] OR (clinical[TIAB] AND pathway[TIAB]) OR (clinical[TIAB] AND pathways[TIAB]) OR (practice[TIAB] AND parameter[TIAB]) OR (practice[TIAB] AND parameters[TIAB]) OR algorithms[MeSH:noexp] OR care pathway[TIAB] OR care pathways[TIAB] OR guidance[TIAB] OR guideline*[TI])

Cochrane Library

#1	muscle injur*
#2	rehabilitation
#3	guidelines
#4	#1 AND #2 AND #3

List of the websites searched:

aspetar.com	(Aspetar, Doha)
ncaa.com	(National Collegiate Athletic Association)

acpsm.com (Association of Chartered Physiotherapists in Sports Medicine)

olympics.com (International Olympic Committee), one record on reporting [https://bjsm.bmj.com/content/54/7/372]

efort.org (European Federation of National Associations of Orthopaedics and Traumatology)

eskka.org (European Society of Sports Traumatology, Knee Surgery and Arthroscopy)

acsm.org (American College of Sports Medicine)

bjsm.bmj.com (British Journal of Sports Medicine)

ismult.com (Italian Society of Muscle, Ligaments and Tendons)

Records excluded at the full-text assessment (doi) and reasons for exclusion:

Treating a different Topic:

- 10.1002/14651858.CD003907.pub5
- 10.1002/14651858.CD005956.pub2
- 10.1002/14651858.CD013042.pub2
- 10.5435/JAAOSGlobal-D-19-00104
- 10.5435/JAAOSGlobal-D-19-00105

Not Rehabilitative Intervention:

- 10.1002/14651858.CD006782.pub2
- 10.1136/bjsports-2021-105371
- 10.1007/s40279-016-0647-1
- 10.1136/bjsm.2010.079822
- 10.1136/bjsm.2009.059980

Target population not respecting the inclusion criteria:

- 10.1093/milmed/usaa207
- 10.1097/JSA.0000000000000130

Not Guidelines:

- 10.1177/23259671241281727
- 10.2519/jospt.2022.0501
- 10.1136/bjsports-2020-102213
- 10.1136/bmjsem-2017-000323
- 10.1249/MSS.0000000000001241
- 10.4085/1062-6050-51.3.08
- 10.1590/1413-785220152305142211
- 10.1007/s12178-015-9262-2
- 10.1136/bjsports-2012-091400
- 10.1249/MSS.0b013e31825a36c6
- 10.1016/j.ptsp.2010.07.003
- 10.3928/01477447-20110922-20
- 10.1016/j.csm.2004.06.006
- 10.5694/j.1326-5377.1984.tb104085.x

Not Retrievable:

- 10.1080/09593985.2023.2226732

Articles retrieved in websites and excluded:

Not Rehabilitative

- 10.1177/2325967120902908
