# Supplementary material for: Rehabilitative Good Practices in the Treatment of Patients with Muscle Injuries
Source: J Clin Med. 2025 Jul 29;14(15):5355. doi: 10.3390/jcm14155355 (PMC12347294; doi:10.3390/jcm14155355)
Supplement: Supplementary file 1 [file jcm-14-05355-s001.zip › jcm-3729831 Supplementary Table S1.pdf]

Supplementary Table S1.

Supplementary Table S1A. Classification of muscle injuries according to Mueller-Wohlfahrt H.W. et al., 2012. [13]

**Lesion**	**Indirect**			
	Non-structural			
**Type**	I. Muscle Fatigue Disorder		II. Neuromuscular disorder	
**Classification**	1A: Fatigue-induced muscle disorder	1B: Delayed-onset muscle soreness (DOMS)	2A:Spine-related neuromuscular muscle disorder	2B: Muscle-related neuromuscular muscle disorder
**Definition**	1.A. Circumscribed longitudinal increase of muscle tone (muscle firmness) due to overexertion, change of playing surface or change in training patterns<br>1.B. More generalised muscle pain following unaccustomed, eccentric deceleration movements.<br>2.A. Circumscribed longitudinal increase of muscle tone (muscle firmness) due to functional or structural spinal/lumbopelvical disorder.<br>2.B. Circumscribed (spindle-shaped) area of increased muscle tone (muscle firmness). May result from dysfunctional neuromuscular control such as reciprocal inhibition			
**Symptoms**	1.A. Aching muscle firmness. Increasing with continued activity. Can provoke pain at rest. During or after activity<br>1.B. Acute inflammative pain. Pain at rest. Hours after activity<br>2.A. Aching muscle firmness. Increasing with continued activity. No pain at rest<br>2.B. Aching, gradually increasing muscle firmness and tension. Cramp-like pain			

**Clinical Signs**	1.A. Dull, diffuse, tolerable pain in involved muscles, circumscribed increase of tone. Athlete reports of 'muscle tightness'<br>1.B. Oedematous swelling, stiff muscles. Limited range of motion of adjacent joints. Pain on isometric contraction. Therapeutic stretching leads to relief<br>2.A. Circumscribed longitudinal increase of muscle tone. Discrete oedema between muscle and fascia. Occasional skin sensitivity, defensive reaction on muscle stretching. Pressure pain<br>2.B. Circumscribed (spindle-shaped) area of increased muscle tone, oedematous swelling. Therapeutic stretching leads to relief. Pressure pain
**Location**	1.A. Focal involvement up to entire length of muscle<br>1.B. Mostly entire muscle or muscle group<br>2.A. Muscle bundle or larger muscle group along entire length of muscle<br>2.B. Mostly along the entire length of the muscle belly
**Ultrasound/MRI**	Ultrasound: often negative; transient hyper- or hypo-echoic changes over time, after 3-5 days; Power Doppler US: negative.<br>MRI: negative; sometimes shows evidence of limited edema.
**Prognosis**	5-15 days.

**Supplementary Table S1B**				
**Lesion**	**Indirect**			
	Structural			
**Type**	III: Partial muscle injury.		IV: (Sub)total muscle injury.	
**Classification**	3A: Minor partial muscle tear	3B: Moderate partial muscle tear	4: (Sub)total muscle tear/tendinous avulsion	

**Definition**	3A: Tear with a maximum diameter of less than muscle fascicle/bundle.<br>3B: Tear with a diameter of greater than a fascicle/bundle<br>4: Tear involving the subtotal/complete muscle diameter/tendinous injury involving the bone–tendon junction<br>CDI: Direct muscle trauma, caused by blunt external force. Leading to diffuse or circumscribed haematoma within the muscle causing pain and loss of motion
**Symptoms**	3A: Sharp, needle-like or stabbing pain at time of injury. Athlete often experiences a ‘snap’ followed by a sudden onset of localised pain<br>3B: Stabbing, sharp pain, often noticeable tearing at time of injury. Athlete often experiences a ‘snap’ followed by a sudden onset of localised pain. Possible fall of athlete<br>4: Dull pain at time of injury. Noticeable tearing. Athlete experiences a ‘snap’ followed by a sudden onset of localised pain. Often fall
**Clinical Signs**	3A: Well-defined localised pain. Probably palpable defect in fibre structure within a firm muscle band. Stretch-induced pain aggravation<br>3B: Well-defined localised pain. Palpable defect in muscle structure, often haematoma, fascial injury Stretch-induced pain aggravation<br>4: Large defect in muscle, haematoma, palpable gap, haematoma, muscle retraction, pain with movement, loss of function, haematoma
**Location**	3.A/B: Primarily muscle–tendon junction<br>4: Primarily muscle–tendon junction or Bone–tendon junction

**Ultrasound/MRI**	3A: Ultrasound: Slightly hyperechoic area that subsequently becomes inhomogeneous and hypoechoic, well-localized, with some structural disorder; a small anechoic area can be detected in the musculature.<br>MRI: Imbibition, edema, and mild homogeneous hyperintense signal for interstitial and peri-fascial edema or small hemorrhagic extravasation.<br>3B: Ultrasound: Hyperechoic area that becomes markedly inhomogeneous, with evidence of structural disorder and a large anechoic area inside and outside the muscle.<br>MRI: The muscle is enlarged due to imbibition, edema, with inhomogeneous hyperintensity related to interstitial and peri-fascial edema or hemorrhagic extravasation.<br>4: Ultrasound: Inhomogeneous and disorganized areas, iso or hyperechoic. Subsequently, inhomogeneity and marked structural changes, retraction of the ends of the torn muscle, and a large anechoic area within the muscle and between the muscles and the muscle ends.<br>MRI: Muscle retraction, hyperintense fluid caused by hemorrhagic extravasation between the two muscle ends.
**Prognosis**	3A: Proximal, central, and distal lesions: 15-18 days.<br>3B: Proximal, central, and distal lesions: 25-35 days.<br>4: ≥ 60 days.

**Supplementary Table S1C**	
**Direct Lesion**	**Contusion Direct injury**
**Definition**	Direct muscle trauma, caused by blunt external force. Leading to diffuse or circumscribed haematoma within the muscle causing pain and loss of motion
**Symptoms**	Dull pain at time of injury, possibly increasing due to increasing haematoma. Athlete often reports definite external mechanism
**Clinical Signs**	Dull, diffuse pain, haematoma, pain on movement, swelling, decreased range of motion, tenderness to palpation depending on the severity of impact. Athlete may be able to continue sport activity rather than in indirect structural injury

**Location**	Any muscle, mostly vastus intermedius and rectus femoris
**Ultrasound/MRI**	Diffuse or circumscribed haematoma in varying dimensions
