# Supplementary material for: Rehabilitative Good Practices in the Treatment of Patients with Muscle Injuries
Source: J Clin Med. 2025 Jul 29;14(15):5355. doi: 10.3390/jcm14155355 (PMC12347294; doi:10.3390/jcm14155355)
Supplement: Supplementary file 1 [file jcm-14-05355-s001.zip › jcm-3729831 Supplementary Table S2.pdf]

Supplementary Table S2: British Athletics Muscle Injury Classification (BAMIC) [14]			
**Grade**	**Suffix**	**Description**	**MRI Features**
0	a	Focal neuromuscular injury with normal MRI	Normal MRI
	b	Generalized muscle soreness with normal MRI or MRI characteristic of DOMS	Normal MRI or MRI with generalized, patchy high signal change affecting several muscles
1	a	**Myofascial:** Small muscle tear extending from the fascia	High signal change on STIR/fat-suppressed images in the periphery of the muscle, no greater than 10% into the muscle and with a longitudinal length of less than 5 cm within the muscle.
	b	**Musculotendinous:** Small muscle tear located within the muscle or at the MTJ	High signal change over a limited area of less than 5 cm and less than 10% of the muscle cross-sectional area at its maximal site.
	c	**Intratendinous:** *There are no Grade 1 injuries in this classification which involve disruption within the tendon.*	
2	a	**Myofascial:** Moderate muscle tear extending from the peripheral fascia into the muscle	High signal change from the periphery of the muscle. The high signal change will either measure between 10% and 50% of the cross-sectional area of that individual muscle at the site of injury or extend between 5 and 15 cm within the muscle.

	b	**Musculotendinous:** Moderate muscle tear occurring within the muscle or at the MTJ	High signal change will either measure between 10% and 50% of the muscle cross-sectional area or have a longitudinal length between 5 and 15 cm.
	c	**Intratendinous:** Moderate muscle tear extending into the tendon	Injury within the tendon is evident over a longitudinal length of less than 5 cm and less than 50% of the maximal tendon diameter on axial images.
3	a	**Myofascial:** Extensive muscle tear extending from the peripheral fascia into the muscle	High signal change patterns of greater than 50% of the muscle cross-sectional area or greater than 15 cm in length. There will be evidence of architectural fibre disruption which is likely to be greater than 5 cm.
	b	**Musculotendinous:** Extensive muscle tear located within the muscle or at the MTJ	High signal change patterns of greater than 50% of the muscle cross-sectional area or greater than 15 cm in length. There will be evidence of architectural fibre disruption which is likely to be greater than 5 cm

	c	**Intratendinous:** Extensive muscle tear extending into the tendon	Evidence of injury in the tendon over a longitudinal length of greater than 5 cm or greater than 50% of the tendon's maximal cross-sectional area. There is no evidence of a complete defect but there may be loss of the usual straight margins and tendon tension suggesting some loss of the tendon integrity.
4		**Complete muscle tear**	Complete tear to the muscle.
		**Intratendinous: Complete tendon tear**	Complete tear to the tendon.

British Athletics Muscle Injury Classification (BAMIC) [12] MTJ: Musculotendinous junction. Grades 1-4 are classified based on the MRI features of the muscle injury. Suffixes "a", "b" and "c" indicate the location of the injury. The classification has been developed primarily for hamstring injuries but could be extrapolated to other muscle injuries.
